# Validity and reliability of the Turkish version of the modified Yale Preoperative Anxiety Scale

**DOI:** 10.3906/sag-1612-113

**Published:** 2019-06-18

**Authors:** Zehra HATİPOĞLU, Oğuzhan KIRDÖK, Dilek ÖZCENGİZ

**Affiliations:** 1 Department of Anesthesiology and Reanimation, Faculty of Medicine, Çukurova University, Adana Turkey; 2 Department of Psychological Counseling and Guidance, Faculty of Education, Çukurova University, Adana Turkey

**Keywords:** Anxiety, validation studies, pediatrics

## Abstract

**Background/aim:**

The modified Yale Preoperative Anxiety Scale (m-YPAS) is widely used to measure children’s anxiety levels. The aim of this study was to translate the m-YPAS into Turkish and test its validity and reliability in Turkish children.

**Materials and methods:**

The English version of the m-YPAS was translated into Turkish using the forward-back-forward translation technique. This study enrolled 120 children. The m-YPAS was administered to 120 children who were recorded on video. The State-Trait Anxiety Inventory for Children (STAIC) was used for only 30 of 120 children. The videotapes were evaluated by two experienced observers [an anesthesiologist, ObA(an), and a psychologist, ObB(ps)]. The interrater reliability, concurrent validity, sensitivity, specificity, and positive and negative predictive values were analyzed.

**Results:**

The mean age of the children was 7.8 ± 2.2 years. The weighted kappa values of the m-YPAS between observers were in substantial agreement (κw = 0.74–0.80) and almost perfect agreement (κw = 0.84–0.85). The Cronbach alpha values were high [α = 0.85 for ObA(an) and α = 0.86 for ObB(ps)]. The correlation between m-YPAS and STAIC showed good agreement (P < 0.05). The sensitivity and specificity were high, and the predictive value was 92.86%.

**Conclusion:**

The Turkish version of the m-YPAS can be applied as a reliable and valid observational questionnaire for Turkish children.

## 1. Introduction

Hospitalization for children, especially for surgery, is a traumatic process. Children may develop anxiety and fear in this environment. Several studies have reported preoperative anxiety in up to 60% of children undergoing surgery and anesthesia [1]. Anxiety is not only important for the preoperative period, but it also has an effect on the postoperative period, because postoperative behavioral changes (e.g., separation anxiety, nightmares, aggression toward authority, nocturnal enuresis) and emergence delirium may develop in children with preoperative anxiety [2–4]. To reduce preoperative anxiety in children, pharmacological and nonpharmacological methods are used. Pharmacological methods involve sedative agents, whereas nonpharmacological methods include the presence of parents, hypnosis, music, distraction techniques, fun transportation systems, acupuncture, and preoperative information programs [5]. 

Several scales have been developed to evaluate the effect of the stress-reducing methods. These scales include the State-Trait Anxiety Inventory for Children (STAIC), the Yale Preoperative Anxiety Scale (YPAS), and the modified Yale Preoperative Anxiety Scale (m-YPAS). The STAIC is a self-reporting questionnaire used as an indicator of anxiety in children, but it is not a practical method due to the age limit (as applicability is for those above 5 years) and the length of the response time (average: 5–10 min) in busy operating environments. Due to these limitations, the Yale Preoperative Anxiety Scale (YPAS) was developed by Kain in 1995. The YPAS is an observational checklist used for children aged 2–6 years, and children’s anxiety is evaluated within a short time during anesthesia induction. Kain et al. then modified it to the m-YPAS in 1997. The reliability and validity of the m-YPAS were shown. For interrater reliability, the values of weighted kappa (κw) ranged from 0.68 to 0.86. For intraobserver reliability, agreement was seen as good to excellent (κw = 0.63–0.90). The correlation between the m-YPAS and STAIC was good. Consequently, they stated that the m-YPAS shows good psychometric properties for assessing children’s anxiety [6]. Unlike the YPAS, the m-YPAS is performed for children aged 5 to 12 years old, both in the preoperative unit and during anesthesia induction, and at present it is widely used to evaluate children’s anxiety before surgery [3,6]. 

The reliability and validity of this scale have been adapted to Swedish, Danish, Spanish, and Korean [7–10]. Proczkowska Björklund et al. reported that the Swedish version of the m-YPAS has good consistency, interrater validity, and construct validity [8]. Similarly, the Korean and Spanish versions of the m-YPAS show good psychometric properties [7,10]. Skovby et al. performed preliminary testing of the Danish version of the m-YPAS, and they emphasized that the Danish version indicates satisfactory face validity and interrater reliability [9].

Anxiety is an important issue in all children, and knowing the anxiety level can prevent some important problems. For this reason, anesthesiologists may arrange their techniques during operation and the postoperative period. Although the m-YPAS is used in different languages around the world, there is a deficiency in Turkish children in this area. Therefore, the present study aims to translate the m-YPAS into Turkish and evaluate its reliability and validity. 

## 2. Materials and methods

After obtaining consent from the developer, Dr Zeev Kain, to use and translate the m-YPAS into Turkish, this study was approved by the local ethics committee (No: 8-42/2015).

### 2.1. Participants

Written informed consent was obtained from the parents. From May 2015 to December 2015, 120 children (average age: 7.8 ± 2.2 years) undergoing elective surgery were enrolled in this study. The sample included 36 females and 84 males. The sample size should be 5 or 10 times the number of items in the scale. According to the literature, the sample size of the study was estimated at 120 patients [11,12]. One hundred and twenty children were recorded on video. The STAIC was performed for only 30 of 120 children. The children who had writing and reading skills and understood the meaning of the sentences (as required for children who will perform the STAIC), and with physical status I–II as defined by the American Society of Anesthesiologists (ASA), were included in the study. Exclusion criteria were as follows: children with psychiatric and neurological disorders, children who had been operated on previously, emergency surgeries, non-Turkish speaking children or parents, and those who refused to participate. All children irrespective of the type of surgery were included in this study. In the preoperative unit, none of the children were given premedication. After children were recorded on video and performed the STAIC, they were then taken into the operating room. All children received general anesthesia. After the end of surgery, they were transported to the recovery room. 

### 2.2. Instruments

The m-YPAS is used to evaluate children’s preoperative anxiety. It contains 22 items in 5 categories (activity, emotional expression, state of arousal, vocalization, and use of parents). Activity, emotional expression, state of arousal, and use of parents all include four items. However, vocalization includes six items. A score of 1 is assigned for each item. The raw score is obtained for each category, and then a partial weight is calculated. The total adjusted score is calculated with the formula (activity / 4 + emotional expression / 4 + the state of arousal / 4 + use of parents / 4 + vocalization / 6) × 100 / 5 [6]. The m-YPAS can be used both in the preoperative unit and at the beginning of anesthesia [13]. In this study, the m-YPAS was only applied in the preoperative unit.

The STAIC is used to assess anxiety in children. It was modified from the State Trait Anxiety Inventory (STAI) used for adults by Spielberger in 1973 [7,14]. The Turkish version of the STAIC was improved by Özusta in 1995 [15]. The STAIC has two components: State-Anxiety (STAIC-S) and Trait-Anxiety (STAIC-T). STAIC-S explains the transitory emotional response at a certain moment, while STAIC-T describes generalized anxiety. Each scale includes 20 items. Response options in the questionnaire are “hardly ever”, “sometimes”, and “very often”. Children answer by checking one of the three options for all items. The options are scored from 1 to 3 (“very often”: 3 points, “hardly ever”: 1 point). Total anxiety scores for each scale range from 20 (minimum) to 60 (maximum) [15]. In our study, STAIC-S was preferred because children experience transient anxiety in the preoperative period.

### 2.3. Procedure 

The original version of the m-YPAS was translated into Turkish and then back into English. The back-translation technique developed by Brislin was used [16]. The forward translation from the original version of the m-YPAS was performed by two native Turkish-speaking translators (an anesthesiologist and a psychologist) (Step I). Consensus on the Turkish version was achieved by an expert group (two anesthesiologists, a psychologist, and a pediatric anesthesiology consultant) (Step II). The Turkish version of the m-YPAS was then translated back into English. This process was done by two English lecturers with Turkish as their mother tongue and no knowledge of the m-YPAS (Step III). The back translation was evaluated in comparison with the two English versions, and a consensus was reached (Step IV). Finally, suitable changes were made by an expert group and the Turkish version of the m-YPAS was accepted for use in this study (Step V). 

One hundred and twenty patients and their parents were recorded on video for 2 min in the preoperative unit. The videotapes were used to evaluate the anxiety levels of children and they were assessed independently by two observers [an anesthesiologist, ObA(an), and an experienced observer psychologist, ObB(ps)]. 

### 2.4. Statistical analysis 

IBM SPSS Statistics 22 (IBM Corp., Armonk, NY, USA) was used for analysis. Descriptive data were expressed as the number of cases (percentage), median, interquartile range, and confidence interval (95%). Nonparametric methods were employed as variables were at an ordinary level of measurement and the data were not accepted to be normally distributed (Kolmogorov–Smirnov test). 

Reliability was measured in two ways: interrater reliability (interobserver) and internal consistency [17]. The interrater reliability was assessed with kappa (κ) and weighted kappa (κw) statistics. The corresponding ranges of kappa are as follows: <0.00 poor agreement, 0.00–0.20 slight agreement, 0.21–0.40 fair agreement, 0.41–0.60 moderate agreement, 0.61–0.80 substantial agreement, and 0.81–1.00 almost perfect agreement, and results were presented according to these ranges [18]. Additionally, the intraclass correlation coefficient (ICC) was used in the agreement between the overall weighted scores because the m-YPAS contains a numerical rating based on observation. The ICC is a statistical analysis used in the agreement of the measurements made by different observers [19]. The acceptable levels of the ICC are as follows: <0.70 incompatible agreement, 0.70–0.84 good (acceptable) agreement, 0.85–0.94 high agreement, and 0.95–1.0 excellent agreement, and results were presented according to these ranges [20]. Cronbach’s alpha (α) was used to measure internal consistency and was expressed as a number between 0 and 1 [21]. Furthermore, the difference in the scores of the observers was evaluated with the Wilcoxon signed-rank test (z).

Spearman’s rank correlation test (rs) was used to assess the correlation between the Turkish version of the m-YPAS and the Turkish version of the STAIC. P < 0.05 was considered statistically significant.

For determining scores that indicate high anxiety in children undergoing surgery in the Turkish version of the m-YPAS, we used the Turkish version of the STAIC as a gold standard. According to Spielberger’s manual, the cutoff score for high anxiety was the mean value plus 1 standard deviation value in the normative group [7]. In this context, the Turkish version of the STAIC provides normative data for children aged 9–12 years. The mean value and standard deviation of the Turkish version of STAIC-S were 31.79 and 6.26, respectively. The cutoff score for high anxiety was 38.05 [15]. In other words, children who scored more than 38.05 on the Turkish version of STAIC-S were defined as high anxiety cases. Later, the sensitivity, specificity, and positive and negative predictive values were analyzed for different cutoff scores in the Turkish version of the m-YPAS in relation to the Turkish version of the STAIC-S by using a receiver operating characteristic (ROC) curve.

## 3. Results

### 3.1. Participant characteristics

One hundred twenty patients were accepted into this study. The sex distribution was 84 (70%) males and 36 (30%) females. The average age of children was 7.8 ± 2.2 years. The patients underwent surgeries such as strabismus, orchidopexy, circumcision, and adenoidectomy.

### 3.2. Interrater reliability

The values of the Turkish m-YPAS for ObA(an) and ObB(ps) were 40.50 ± 15.28, 40.78 ± 14.84, respectively, and that for the Turkish STAIC was 37.77 ± 8.80 (Table 1). The reliability analysis results from video recordings evaluated by two independent observers are presented in Table 2. The weighted kappa values of the m-YPAS between ObA(an) and ObB(ps) were in substantial agreement (activity κw = 0.74, emotional expressivity κw = 0.76, and state of apparent arousal κw = 0.80) and almost perfect agreement (vocalizations κw = 0.84 and use of parents κw = 0.85). The ICC values were in high agreement (0.88 for activity, 0.90 for emotional expressivity, 0.93 for state of arousal) and excellent agreement (0.95 for vocalizations, 0.95 for use of parents) (Table 3). Cronbach’s alpha values in terms of internal consistency were higher at α = 0.85 for ObA(an) and α = 0.86 for ObB(ps) (Table 4). According to the results of the Wilcoxon signed-rank test, no difference was found between the assessments of the observers (P > 0.05) (Table 5). 

**Table 1:** Descriptive statics of the Turkish versions of the m-YPAS and STAIC for ObA(an) and ObB(ps).

	Observers	n	Mean	SD
Activity	ObA(an)	120	8.50	3.81	ObB(ps)	120	8.45	3.29
	Vocalizations	ObA(an)	120	5.58	3.02	ObB(ps)	120	5.94	3.18
Emotional expressivity		ObA(an)	120	10.16	4.10	ObB(ps)	120	10.21	3.96
	State of apparent arousal	ObA(an)	120	8.88	4.17	ObB(ps)	120	8.75	4.16
Use of parents		ObA(an)	120	7.37	3.94	ObB(ps)	120	7.41	3.77
	Total m-YPAS	ObA(an)	120	40.50	15.28	ObB(ps)	120	40.78	14.84
STAIC			30	37.77	8.80

m-YPAS, Modified Yale Preoperative Anxiety Scale. STAIC, State-Trait Anxiety Inventory for Children. ObA(an), Native observer anesthesiologist. ObB(ps), Experienced observer psychologist. SD, Standard deviation.

**Table 2:** Kappa values of the Turkish m-YPAS between observers.

	k	kW	kW/CI	Percentage agreement (%)
Activity	0.71	0.74*	0.63–0.84	82.5
Vocalizations	0.79	0.84α	0.77–0.92	86.7
Emotional expressivity	0.71	0.76*	0.67–0.85	80.8
State of apparent arousal	0.74	0.80*	0.72–0.88	83.3
Use of parents	0.78	0.85α	0.77–0.92	89.2

*Substantial agreement, αAlmost perfect agreement. CI, Confidence interval. m-YPAS, Modified Yale Preoperative Anxiety Scale.

**Table 3:** Intraclass correlation coefficient of the Turkish m-YPAS between observers.

	ICC	ICC/CI	P
Activity	0.88	0.82–0.91	0.000
Vocalizations	0.95	0.93–0.97	0.000
Emotional expressivity	0.90	0.85–0.93	0.000
State of apparent arousal	0.93	0.89–0.95	0.000
Use of parents	0.95	0.93–0.97	0.000
Total m-YPAS	0.97	0.96–0.98	0.000

ICC, Intraclass correlation coefficient. CI, Confidence interval. m-YPAS, Modified Yale Preoperative Anxiety Scale.

**Table 4:** Cronbach’s alpha values.

	α	CI
ObA(an)	0.85	0.80–0.84
ObB(ps)	0.86	0.83–0.84

ObA(an), Native observer anesthesiologist. ObB(ps), Experienced observer psychologist. α, Cronbach’s alpha; CI, confidence interval.

**Table 5:** z values of the Turkish m-YPAS between ObA(an) and ObB(ps).

	Groups	n	x̄rank	Ʃrank	z	P
Activity	Negative ranks	10	12.30	103.00	–0.21a	0.834
ObA(an) – ObB(ps)	Positive ranks	11	10.00	68.00		
	Ties	99				
	Total	120				
Vocalizations	Negative ranks	11	8.55	94.00	–0.76b	0.448
ObA(an) –ObB(ps)	Positive ranks	10	13.70	137.00		
	Ties	99				
	Total	120				
Emotional expressivity	Negative ranks	12	11.00	132.00	–0.20b	0.840
ObA(an) – ObB(ps)	Positive ranks	11	13.09	144.00		
	Ties	97				
	Total	120				
State of apparent arousal	Negative ranks	12	10.00	120.00	- 0.63a	0.532
ObA(an) – ObB(ps)	Positive ranks	8	11.25	90.00		
	Ties	100				
	Total	120				
Use of parents	Negative ranks	6	7.00	42.00	- 0.28b	0.782
ObA(an) – ObB(ps)	Positive ranks	7	7.00	49.00		
	Ties	107				
	Total	120				
Total m-YPAS	Negative ranks	27	28.48	769.00	- 0.47b	0.641
ObA(an) – ObB(ps)	Positive ranks	30	29.47	884.00		
	Ties	63				
	Total	120				

z, Wilcoxon signed-rank. a Based on positive ranks, b based on negative ranks. ObA(an), Native observer anesthesiologist. ObB(ps), Experienced observer psychologist.

### 3.3. Concurrent validity

The result of Spearman’s rank correlation test (rs) between the Turkish versions of the m-YPAS and STAIC was high (rs = 0.76 and P = 0.000 for ObA(an), and**rs = 0.85 and P = 0.001 for ObB(ps)) (Table 6).

**Table 6:** The correlation between the Turkish versions of the m-YPAS and STAIC for ObA(an) and ObB(ps).

	n	ObA(an)		ObB(ps)	
		rs	P	rs	P
Activity	30	0.55	0.002	0.70	0.000
Vocalizations	30	0.43	0.019	0.55	0.002
Emotional expressivity	30	0.71	0.000	0.62	0.000
State of apparent arousal	30	0.47	0.009	0.59	0.001
Use of parents	30	0.49	0.007	0.57	0.001
Total m-YPAS	30	0.76	0.000	0.85	0.000

rs, Spearman’s rank correlation. ObA(an), Native observer anesthesiologist. ObB(ps), Experienced observer psychologist. m-YPAS, Modified Yale Preoperative Anxiety Scale. STAIC, State-Trait Anxiety Inventory for Children.

### 3.4. ROC analysis

The reference score for high anxiety was 38.05 according to the study of the Turkish version of STAIC-S. The cutoff score of the Turkish version of the m-YPAS is 40, the sensitivity and specificity are high, and the predictive value is 92.86% (Table 7). At this score, only four patients (13.33%) were misclassified: one false positive and three false negatives. The area under the curve (AUC) was 0.904 (0.790–1.000, P < 0.0001) (Figure). 

**Table 7:** Sensitivity, specificity, positive predictive value, negative predictive value, and accuracy.

Cutoff score	Sensitivity (%)	Specificity (%)	PPV (%)	NPV (%)	Accuracy (%)
35.00	93.75	50.00	68.18	87.50	73.33
37.50	81.25	85.71	86.67	80.00	83.33
40.00	81.25	92.86	92.86	81.25	86.67
42.50	62.50	100.00	100.00	70.00	80.00

Data are expressed as percentages. PPV, Positive predictive value. NPV, Negative predictive value.

**Figure:**
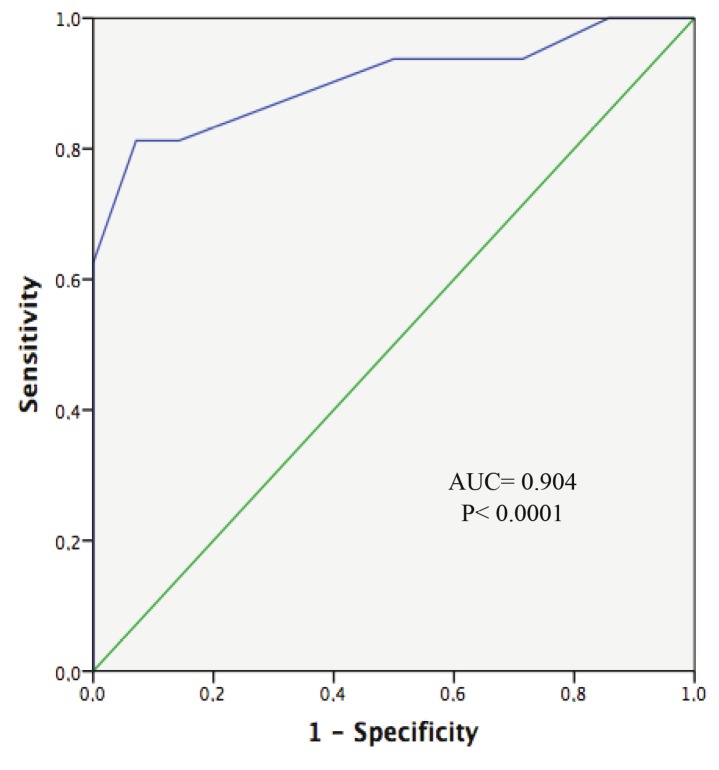
Receiver operating characteristic (ROC) curve for high anxiety. AUC: Area under the curve.

## 4. Discussion

At present, the m-YPAS scale is used to evaluate preoperative anxiety levels in children. In this study, the English version of the m-YPAS has been adapted to Turkish with both reliability and validity tests. 

Jung et al. reported that internal consistency and test-retest reliability (intraobserver) were used to assess the reliability of the Korean m-YPAS. One hundred two children were evaluated by an experienced child and adolescent psychiatrist, surgeon, and nurse in their study. The test-retest reliability and Cronbach’s α values were 0.74 (0.64–0.85) and 0.93, respectively. Consequently, they explained that the Korean m-YPAS is a useful tool for assessing preoperative anxiety [7]. The test-retest method is one of the reliability analyses, but there may be several drawbacks to this method, such as memorizing risk and application effect [17]. Therefore, interrater reliability as the statistical analysis was preferred in our study. The weighted kappa values of “activity” and “emotional expressivity” categories were slightly lower than other categories of the m-YPAS. The reason for this difference could be the evaluations of observers who have different professional experiences. However, we stated that the results of the interrater reliability measurement and internal consistency were high. Likewise, the ICC value was in excellent agreement in our study. These results are consistent with the original, the Spanish, and the Swedish versions of the m-YPAS [6,8,10]. According to these results, we would like to state that the Turkish version of the m-YPAS is a reliable assessment tool between observers. 

In a validation study with two phases by Proczkowska Björklund et al., 52 patients were analyzed by two student registered nurse anesthetists (SRNAs) and a certified registered nurse anesthetist (CRNA) in phase 1. The videotapes of 98 patients were evaluated by two CRNAs in phase 2. Their results showed that weighted kappa values in phase 2 were higher than phase 1 and this result was explained as an effect of having experienced persons in pediatric anesthesia [8]. The reliability of the scale was assessed independently by two experienced observers in this study, and the agreement between observers was perfect. We believe that the experience of an observer may contribute to minimizing unexpected results. The Turkish version can be safely used by different researchers (Appendix). 

The applicability of a scale is dependent not only on the reliability but also the validity. Previously, the STAIC was the gold standard for assessing anxiety in children. Hence, concurrent validity was measured by the correlation between the Turkish versions of the m-YPAS and STAIC. The results of the “vocalization” and “use of parents” categories were slightly lower than those of other categories. We would like to emphasize that the m-YPAS is performed by an observer while the STAIC is a self-reporting questionnaire. In light of this information, we explained that the person may be insufficient in assessing his or her own behavior. This situation may cause a decrease in the correlation. However, the result of the correlation between the Turkish versions of the m-YPAS and STAIC was quite strong in this study. These results are consistent with the original version of the m-YPAS [6]. In the Swedish version of the m-YPAS, Proczkowska Björklund et al. used the a numeric analogue scale (NAS) for concurrent validity. The children’s anxiety was evaluated using the NAS scale by a parent, a CRNA, and SRNAs in phase 1 of their study. They reported that the values of rs were slightly low than other values, and that was explained by inexperience about pediatric anesthesia [8].

As a reference point, a score of 37 on the STAIC was chosen by Kain et al. Similarly, Jung et al. used a score of 45 on the Korean version of the STAIC-S [7]. For this study, the normative data were obtained from the Turkish version of STAIC-S, and this value was 38 [15]. However, this self-questionnaire has good reliability and validity data for children aged 9–12 years. In our study, 30 children aged 8–12 years were included for determining the scores of the Turkish version of the m-YPAS that indicate high anxiety. Consequently, the sensitivity, specificity, and positive and negative predictive values pointed to good results. However, it should be noted that when evaluated together with the validation results described above, the Turkish version of the m-YPAS was only appropriately validated for children aged 8–12 years. There is no gold standard for anxiety evaluation in children less than 8 years of age. The cutoff score of the m-YPAS for these children may be lower than children aged 8–12 years [15].

There are some limitations to this study. First, in the validation of the Turkish version of the m-YPAS against the Turkish version of STAIC-S, lack of participants for various reasons can be considered as a limitation of this study. Second, the m-YPAS was only applied in the preoperative period in this study. Because it can be used both in the preoperative unit and at induction of anesthesia, construct validity was not established.

This study has indicated the psychometric properties of the Turkish version of the m-YPAS for children aged 5–12 years. This tool can be used to evaluate preoperative anxiety in children by different investigators. In conclusion, the Turkish version of the m-YPAS can be used as a reliable and valid tool in future studies.
